# MMP1, MMP9, and COX2 Expressions in Promonocytes Are Induced by Breast Cancer Cells and Correlate with Collagen Degradation, Transformation-Like Morphological Changes in MCF-10A Acini, and Tumor Aggressiveness

**DOI:** 10.1155/2013/279505

**Published:** 2013-05-09

**Authors:** G. K. Chimal-Ramírez, N. A. Espinoza-Sánchez, D. Utrera-Barillas, L. Benítez-Bribiesca, J. R. Velázquez, L. A. Arriaga-Pizano, A. Monroy-García, E. Reyes-Maldonado, M. L. Domínguez-López, Patricia Piña-Sánchez, E. M. Fuentes-Pananá

**Affiliations:** ^1^Unidad de Investigación Médica en Enfermedades Infecciosas y Parasitarias, Hospital de Pediatría, Centro Médico Nacional Siglo XXI, Instituto Mexicano del Seguro Social (IMSS), Avenida Cuauhtémoc 330, Colonia Doctores, Delegación Cuauhtémoc, 06720 México City, DF, Mexico; ^2^Programa de Maestría en Ciencias Quimicobiológicas, Escuela Nacional de Ciencias Biológicas, Instituto Politécnico Nacional (IPN), Prolongación de Carpio y Plan de Ayala, Casco de Santo Tomás, 11340 México City, DF, Mexico; ^3^Unidad de Investigación Médica en Enfermedades Oncológicas, Hospital de Oncología, Centro Médico Nacional Siglo XXI, Instituto Mexicano del Seguro Social (IMSS), Avenida Cuauhtémoc 330, Colonia Doctores, Delegación Cuauhtémoc, 06720 México City, DF, Mexico; ^4^Laboratorio de Inmunoalergia y Asma, Instituto Nacional de Enfermedades Respiratorias (INER), Calzada de Tlalpan 4502, Colonia Belisario Domínguez Sección, Delegación Tlalpan, 14080 México City, DF, Mexico; ^5^Unidad de Investigación Médica en Inmunoquímica, Hospital de Especialidades, Centro Médico Nacional Siglo XXI, Instituto Mexicano del Seguro Social (IMSS), Avenida Cuauhtémoc 330, Colonia Doctores, Delegación Cuauhtémoc, 06720 México City, DF, Mexico; ^6^Departamento de Morfología de la Escuela Nacional de Ciencias Biológicas, Instituto Politécnico Nacional (IPN), Prolongación de Carpio y Plan de Ayala, Casco de Santo Tomás, 11340 México City, DF, Mexico; ^7^Departamento de Inmunología de la Escuela Nacional de Ciencias Biológicas, Instituto Politécnico Nacional (IPN), Prolongación de Carpio y Plan de Ayala, Casco de Santo Tomás, 11340 México City, DF, Mexico

## Abstract

Tumor-associated immune cells often lack immune effector activities, and instead they present protumoral functions. To understand how tumors promote this immunological switch, invasive and noninvasive breast cancer cell (BRC) lines were cocultured with a promonocytic cell line in a Matrigel-based 3D system. We hypothesized that if communication exists between tumor and immune cells, coculturing would result in augmented expression of genes associated with tumor malignancy. Upregulation of proteases *MMP1* and *MMP9* and inflammatory *COX2* genes was found likely in response to soluble factors. Interestingly, changes were more apparent in promonocytes and correlated with the aggressiveness of the BRC line. Increased gene expression was confirmed by collagen degradation assays and immunocytochemistry of prostaglandin 2, a product of COX2 activity. Untransformed MCF-10A cells were then used as a sensor of soluble factors with transformation-like capabilities, finding that acini formed in the presence of supernatants of the highly aggressive BRC/promonocyte cocultures often exhibited total loss of the normal architecture. These data support that tumor cells can modify immune cell gene expression and tumor aggressiveness may importantly reside in this capacity. Modeling interactions in the tumor stroma will allow the identification of genes useful as cancer prognostic markers and therapy targets.

## 1. Introduction

Studies on cancer biology have widely focused on neoplastic cells to understand tumor initiation and progression events [1]. Genes and their molecular pathways contributing to tumor growth have been singled out allowing for the intelligent design of targeted therapies that have increased the overall survival rate in specific neoplasia. However, due to the broad spectrum of triggering mutations, there has been a limited use for such therapies.

More recently, the inflammatory microenvironment in which the tumor develops has also been found to be critical for tumor growth. A handful of cell types constitute the tumor microenvironment and their interactions with the tumor cells are key determinants of malignant progression [2]. Among them, immune cells importantly populate most solid tumors and their functions favor the establishment of local immunosuppression, promote local invasion, and metastasis and allow the appearance of clones resistant to treatment. In breast tumors (BRC), macrophages are found throughout the stroma but are particularly enriched in the invasive front and in the vascular areas of the tumor, in which they may promote tumor invasion and metastasis [3]. In agreement, a meta-analysis showed that in >80% of patients an elevated macrophage density in tumors correlated with poor prognosis [4]. 

Two types of macrophages have been described: M1 or classically activated (by Th1 cytokines) and M2 or alternatively activated (by Th2 cytokines) [5]. M2 macrophages are important suppressors of innate and adaptive immune responses and in homeostatic conditions participate in tissue maintenance, increasing cell proliferation and survival and tissue angiogenesis [6]. M2 macrophages are particularly enriched in aggressive BRCs [7], supporting a model in which the inflammatory tumor microenvironment induces polarization of recruited monocytes into M2 macrophages, thus strengthening protumoral conditions [8]. In agreement, knockout mice for the primary tumor macrophage chemoattractant, CSF-1, have a slow tumor growth and reduced metastasis [9, 10], and CSF-1 levels have been associated with poor prognosis in several human malignancies [11].

It is presently unclear how tumor and stromal cells communicate to establish the inflammatory but tumor promoting conditions. Inflammatory mediators and inflammatory targets with protumor activities have been described, and among the most consistently found in BRC are the following: cyclooxygenase2 (COX2), which is overexpressed in aggressive forms of BRC [12, 13]; CXC chemokine receptor type 4 (CXCR4, also known as fusin or CD184), a potent chemoattractant of lymphocytes and a prognostic marker in BRC [14, 15]; integrin *α*4*β*1 (also termed very late antigen 4, VLA-4), which is enriched in tumor cells with migratory capacities [16]; osteopontin (OPN), also a promoter of cell migration, resistance to apoptosis, increased proteolysis, and vascular regeneration [17, 18]; proteases like metalloproteases 1, 2, and 9 (MMP1, 2, and 9), urokinase plasminogen activator (uPA), and cathepsin D and B, which degrade the extracellular matrix (ECM) promoting tumor invasion and metastasis [19–26]. Tumor supportive mechanisms are likely more limited and common across many types of tumors, and their understanding may help to identify highly effective targets for cancer therapy.

Three-dimensional (3D) cell culture systems have been useful to study cell to cell interactions facilitating tumor growth in BRC. These systems reproduce the mammary gland architecture while allowing manipulation of the microenvironment in which they form [27–30]. In addition to cancer models, Debnath and colleagues developed a 3D culture system of MCF-10A cells, a nontransformed mammary epithelial cell line obtained from a patient with fibroadenoma [31, 32]. MCF-10A cells have been widely used to study the transforming mechanisms of viral and cellular oncogenes; the mammary epithelial acini-like structures they form in Matrigel-based 3D systems have been helpful to understand how oncogenes deregulate processes linked to transformation, such as proliferation and resistance to apoptosis [33, 34].

In order to better understand the communication between tumor cells and immune cells we cocultured poorly and highly invasive BRC cell lines with a promonocyte line in a 3D Matrigel-based system. We hypothesized that if communication exists between tumor cells and promonocytes, their interactions would result in augmented expression of genes associated with tumor malignancy. We found that expressions of proteases *MMP1* and *MMP9* and inflammatory gene *COX2* were favored in coculture conditions. Interestingly, changes were more evident in the monocytic cell line and correlated with the aggressiveness of the BRC line. We confirmed the elevated expression of proteases in collagen degradation assays and with immunocytochemical analysis of prostaglandin 2 (PGE2), a product of COX2 activity. We then used the MCF-10A cells as a sensor of soluble factors with protumoral activities, finding that the acini-like structures formed in the presence of supernatants of the highly aggressive BRC and promonocytes cocultures were of increased size and without well-defined lumens, which often exhibited total loss of the normal architecture. Modeling stromal tumor interactions will allow the identification of genes useful as prognostic markers and therapy targets.

## 2. Material and Methods

### 2.1. Cell Lines and Harvesting of Supernatants

All cell lines were obtained from the American Type Culture Collection (ATCC, Manassas, VA, USA) and culture media and supplements from Gibco BRL Life Technologies (Grand Island, NY, USA) unless specified. MCF-7 cells (No. HTB-22) were cultured in D-MEM/F-12 medium supplemented with 10% heat inactivated, endotoxin-free fetal bovine serum (FBS), 2.5 mM L-glutamine, 14.3 mM sodium bicarbonate, 17.5 mM D-glucose, 15 mM HEPES, and 0.5 mM sodium pyruvate. MDA-MB-231 cells (No. HTB-26) were cultured in Leibovitz's L-15 medium supplemented with 10% FBS, 2 mM of L-glutamine, 0.14M sodium chloride, 5 mM D+ galactose, and 5 mM sodium pyruvate. U937 cells (No. CRL-1593.2) were cultured in RPMI 1640 medium supplemented with 10% FBS, 2 mM glutamine, 23.8 mM sodium bicarbonate, and 11.1 mM D-glucose. MCF-10A cells (No. CRL-10317) were cultured in D-MEM/F-12 medium supplemented with 20 ng/mL of epidermal growth factor (PeproTech, Rocky Hill, NJ, USA), 10 *μ*g/mL insulin, 0.5 *μ*g/mL hydrocortisone, 100 ng/mL cholera toxin (all from Sigma Chemical Co., St. Louis, MO, USA), and 5% fetal horse serum (FHS). All cell cultures also contained 100 units/mL penicillin and 100 *μ*g/mL streptomycin. To obtain supernatants of cell lines in culture, 400,000 cells were seeded in 75 cm^2^ flasks and after 48 h the culture media were recovered, aliquoted, and stored at −20°C. Because U937 cells grow in suspension, they were first centrifuged at 2,000 rpm for 8 min. Each experiment was carried out in triplicate utilizing independent harvests of supernatant.

### 2.2. Cell Labeling

MCF-7 and MDA-MB-231 cells were labeled with CellTracker Red and U937 with CellTracker Blue (both from Molecular Probes, Invitrogen, Carlsbad, CA, USA) before coculturing, to be able to independently sort them after culture. For ECM degradation and cell colocalization analysis U937 cells were labeled with CellTracker Orange. Labeling was done according to the manufacturer's recommended protocols.

### 2.3. Three-Dimensional Coculture Systems

The 3D culture system used was a modification of the Debnath and Sameni overlay method [31, 35, 36]. To analyze mRNA and protein expression, cells were cultured as follows: 10 *μ*L containing 2.5 × 10^5^ cells/well (MCF-7 or MDA-MB-231) in single cell suspensions were seeded in 4-well plates (Lab-Tek Chamber Slide System; Nalge Nunc International, Rochester, NY, USA) on 55 *μ*L of a solidified layer of Matrigel Basement Membrane Matrix (BD Biosciences, San Jose, CA, USA); after 15–20 min, a suspension of 1.25 × 10^5^ U937 cells in 40 *μ*L assay medium (supplemented RPMI 1640 with 60% Matrigel) was added. Finally, a volume of 2 mL of a 1 : 1 mix of D-MEM/F-12 or Leibovitz's L-15 and RPMI 1640 media were added. Cells were harvested either after 4 h for mRNA or after 24 h for protein analysis. Each assay was performed in triplicate and 3D single MCF-7, MDA-MB-231, and U937 cell cultures were included as controls.

### 2.4. Cell Sorting

3D cocultured cells were recovered using a solution of 0.1% trypsin and 0.25% EDTA in phosphate-buffered solution (PBS) and incubated for 3 h at 37°C. Medium with 10% FBS was added to neutralize the trypsin and cells were recovered after centrifugation at 2,000 rpm for 5 min. Recovered cells were washed twice with PBS and then resuspended in PBS with 20% FBS. Individual populations were obtained after sorting (FACS Aria Cell Sorter, Becton & Dickinson Biosciences, San Jose, CA, USA), with purities of at least 95% and viability of >90%.

### 2.5. Real Time (RT-) PCR

Total RNA was extracted from 5 × 10^5^ cells using the RNeasy Mini Kit and 500 ng of RNA were used for cDNA synthesis with the QuantiTect Whole Transcriptome Kit. Both kits were from Qiagen (Hilden, Germany) and protocols were performed as recommended. 

RT-PCRs were performed in capillaries in a 20-*μ*L reaction mix containing LightCyclerTaqman Master (Roche Applied Science, Indianapolis, IN, USA), 0.2 *μ*M specific primer mix, 0.1 *μ*M of gene-specific hydrolysis probes from the Universal Probe Library (Roche), and 2.5 *μ*L of a 1 : 2 or 1 : 10 dilution of cDNA. PCR conditions consisted of 10 min of a preincubation step at 95°C followed by 45 cycles of denaturation (95°C, 10 sec), annealing (60°C, 30 sec), and extension (72°C, 1 sec), followed by a final cooling step of 30 sec at 40°C. The working dynamic range of each gene was determined. Data were normalized to glyceraldehyde 3-phosphate dehydrogenase (GAPDH) levels and the LightCycler (Roche Applied Science) software system was used to analyze the amplified transcripts according to the Cycle threshold (Ct) method. Relative expression was calculated utilizing the Delta-Delta Cycle threshold (DDCt) method.

The primer sequences used were the following: for MMP1, left gctaacctttgatgctataactacga and right tttgtgcgcatgtagaatctg; MMP2, left ataacctggatgccgtcgt and right aggcacccttgaagaagtagc; MMP9, left: gaaccaatctcaccgacagg and right gccacccgagtgtaaccata; uPA, left ttgctcaccacaacgacatt and right ggcaggcagatggtctgtat; COX2, left cttcacgcatcagtttttcaag and right tcaccgtaaatatgatttaagtccac; epiregulin, left aggatggagatgctctgtgc and right ggactgcctgtagaagatgga; CXCR4, left cctctgaggggatcgagtg and right tccccctcaaacccaaag; E-cadherin, left cccgggacaacgtttattac and right gctggctcaagtcaaagtcc, subunit alpha4 of VLA-4 integrin: left ggaatatccagtttttacacaaagg and right agagagccagtccagtaagatga; osteopontin, left gagggcttggttgtcagc and caattctcatggtagtgagttttcc; and GADPH, left caagggcctggtagacaagt and right ctggccctcgtagcacac.

### 2.6. Immunocytochemistry

30,000 sorted cells were centrifuged on positively charged slides (Biocare Medical, Newport Beach, CA, USA) and fixed with an acetone:methanol 1 : 1 mix at −20°C for 10 min. Cells were then hydrated with PBS and permeabilized with 0.05% triton X-100 for 10 min at 4°C. Endogenous peroxidase activity was blocked by incubating the slides in peroxidase blocking solution (Dako, Inc., Carpinteria, CA, USA). Nonspecific antibody binding was blocked by incubation with 8% albumin in PBS for 20 min. The slides were incubated with the following primary antibodies against COX2 (mouse monoclonal anti-human COX2, clone CX-294; DAKO), PGE2 (rabbit polyclonal anti-human PGE2, ab2318; Abcam, Cambridge, UK), or MMP9 (mouse monoclonal anti-human MMP9, 1M37T; Calbiochem, Beeston, Nottinghamshire, UK). No immunized mouse IgG was utilized as negative control (normal mouse IgG; Santa Cruz Biotechnology, Santa Cruz, Ca, USA). Cells were incubated overnight in a moist chamber at 4°C; the dilution for each antibody was 1 : 50. The EnVision Detection Kit (Dako) was employed as the detection system. Cells were counterstained with methylene blue, left to dry at room temperature and permanently coverslipped. Slides were analyzed and photographed with an Olympus BX-41 microscope (Olympus Corporation, Tokyo, Japan). Immunocytochemistry staining intensity was quantified using the Image Pro Plus software, and the integrated optical density (IOD) of 100 cells was obtained.

### 2.7. Collagen Degradation

3D cell cocultures were carried out as described before, but Matrigel was mixed with type IV collagen labeled with fluoresceinisothiocyanate (FITC) (40 *μ*L of Matrigel containing 32.5 *μ*g/mL DQ-collagen IV) (Molecular Probes, Invitrogen, Carlsbad, CA, USA) and cultures were done in BD BioCoat PDL 35 mm coverslip bottom dishes (Becton & Dickinson, Biosciences, San Jose, CA, USA). The final concentration of labeled collagen IV in the Matrigel was 0.5%; this concentration was selected, after concentrations of 0.5%, 1.5%, and 3% were tested. To test for cell migration, after Matrigel degradation cell lines were placed in independent layers of Matrigel and left to polymerize for 15 min. Fluorescence emission was quantified in a Zeiss LSM 510 confocal scanning microscope (Carl Zeiss, Jena, Germany) and data is presented as IOD/50*μ* ^2^; 3D projections were digitally reconstituted from stacks of confocal optical slices of 2-*μ*M. Analysis was done after 5 days in culture.

### 2.8. Analysis of MCF-10A Acinar Structures

MCF-10A acini were formed as described by Debnath et al. [31] with small modifications: a 40 *μ*L base of Matrigel was added per 0.8 cm^2^ well (8-well plates, Lab-Tek Chamber Slide System, Nalge Nunc International, Rochester, NY, USA), incubated for 30 min at 37°C and 800 cells were added in 400 *μ*l of culture medium supplemented with 4 ng/ml of EGF and 2% Matrigel. To analyze acini morphological changes triggered by soluble factors, MCF-10A cells were grown in the presence of supernatant (MCF-10A normal culture medium diluted for 0, 1, 2, 4, 8, or 16 times with supernatant from BRC and promonocyte cell lines, either single or in coculture). Supernatant was replaced every 48 h under same initial conditions; acini morphological changes were monitored every 24 h for 15 days with an optical microscope (IROSCOPE, CA, USA).

### 2.9. Statistical Analysis

All data were analyzed utilizing the SPSS ver 15.0 for Windows statistical software package (SPSS, Inc., Chicago, IL, USA). For acini analysis, one-way analysis of variance (ANOVA) followed by Turkey's *post hoc* test was used. The remaining assays were analyzed by the Kruskal-Wallis test. A value of *P* = 0.05 was considered statistically significant.

## 3. Results

### 3.1. Aggressive Breast Cancer Cells Promote Gene Expression Changes in Promonocytes

**Figure 1:**
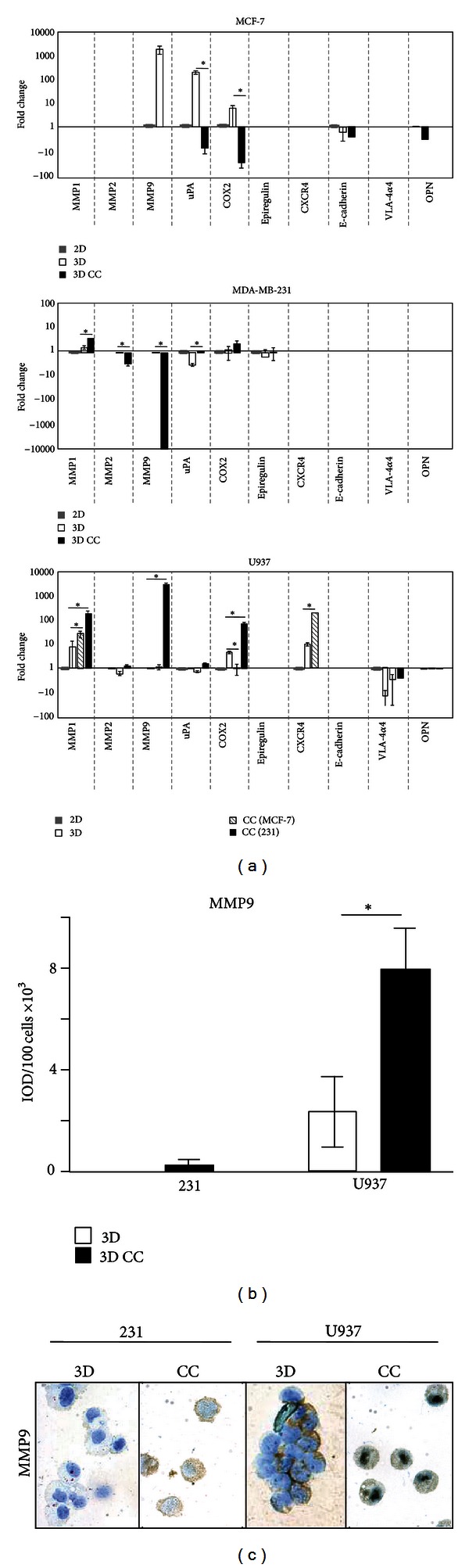
Expression of genes associated with tumor malignancy. (a) Breast cancer cells (MCF-7 and MDA-MB-231) and promonocytes (U937) were cultured individually in monolayer (2D) or in a Matrigel base (3D) or cocultured (CC) in BRC and monocyte pares in the 3D system. Cell lineages were independently isolated and gene expression was measured by RT-PCR. Fold changes are presented related to the 2D expression; when there was not detectable expression in 2D, 3D single cultures served as the basal expression (*OPN* for MCF-7, *MMP2* for MDA-MB-231 and U937, and *MMP9* for U937). Higher and lower expression than basal are marked as positive or negative numbers, respectively. CC (MCF-7) and CC (231) indicate U937 cells isolated from cocultures with MCF-7 or MDA-MB-231 cells. (b) Immunocytochemistry of MMP9 levels. Intensity of signal was quantified for 100 cells for each experiment and the average value is presented. (c) Representative images of MMP9 levels in MDA-MB-231 and U937 cells after single cultures or cocultured together. Bars with asterisks represent comparisons with statistical significant differences (*P* < 0.05). Results are from three independent experiments and only relevant comparisons are presented (CC against 3D and U937/MDA-MB-231 CC against U937/MCF-7 CC).

Considering that many malignant characteristics of tumors result from interactions between tumor and immune cells in the tumor microenvironment, BRC and promonocyte cells were cocultured and changes in gene expression were analyzed. Two BRC epithelial cell lines were used, one poorly aggressive and one highly aggressive, derived from pleural effusions of BRC patients. MCF-7 cells are characterized by a weak invasive capacity and express epithelial markers; MDA-MB-231 cells present a high capacity for invasion and metastasis and accordingly express fibroblastoid mesenchymal markers [37]. Epithelial cells are firmly anchored to the basement membrane, and gaining of mesenchymal cell markers coincides with the acquisition of cell mobility towards external mucosal layers and other organs [38]. Each one of these cells was cocultured in a Matrigel-based 3D system with U937 cells, a promonocyte line derived from a patient with a diffuse histiocytic lymphoma. U937 cells are immature cells of the myelomonocytic lineage [39], which have been a model study of differentiation into mature monocyte/macrophages [40]. Cells in cocultures were individually isolated and changes in expression of genes frequently referred to as markers of cancer malignancy were measured by RT-PCR. Gene expression was compared between cocultured cells and that of individually cultured in monolayer (2D) or in 3D when the 2D culture had null expression (Figure 1(a)).

In MCF-7 cells, null or only basal expression was detected for genes* MMP1*, *MMP2*, *Epiregulin*, *CXCR4*, *E-cadherin,* and the *α4 *subunit of *VLA-4. MMP9* and uPA were over-expressed in MCF-7 single 3D cultures probably as response to interactions with ECM components of the Matrigel layer. However, these genes plus *COX2* and *OPN* were downregulated in coculture conditions. Thus, in the poorly aggressive BRC cell line interactions with promonocytes seem to lower the expression of markers of tumor malignancy (upper panel).

In MDA-MB-231 cells, null or basal levels of expression were found for uPA, *COX2*, *Epiregulin*, *CXCR4*, *E-cadherin*, *α4* subunit of *VLA-4* integrin, and *OPN*. *MMP2* and *MMP9* expressions were downregulated in coculture, and only *MMP1* expression was significantly increased (4.1-fold) when the highly aggressive cell line was cocultured with promonocytes (middle panel).

When gene expression was measured in promonocytes, null or basal levels were found for *MMP2*, uPA, *Epiregulin*, *E-cadherin,* and *OPN*, while lower than basal (2D) expression was found for the *α4 *subunit of *VLA-4* integrin under all 3D conditions. Supporting our initial argument, expression of *CXCR4* increased 19-fold in single 3D cultures and 811-fold when U937 and MCF-7 cells were cocultured. Furthermore, coculture with the aggressive BRC line increased gene expression of *MMP1*, *MMP9,* and *COX2*. *MMP9* (27,270-fold change with respect to the 3D basal condition) and *COX2* (234-fold change) augmented expressions in U937 cells were specific of interacting with the highly aggressive cell line, while *MMP1 *increased 13.9 times at the 3D baseline level, 68 times in coculture with MCF-7, and 772 times with the highly aggressive BRC. In summary, this experiment shows that gene expression changes are promoted when tumor epithelial and promonocyte cells are cocultured together, either by direct interactions or through secreted molecules. With the exception of *MMP1*, most changes in BRC cells have a downward trend, while most upward changes were found in the promonocytic cell line. Of note, those expression changes were significantly higher in coculture with the most aggressive BRC line (Figure 1(a), lower panel).

Significant changes were observed in *MMP9* gene when highly aggressive epithelial tumor and promonocyte cells were cocultured; however those changes are downward in epithelia and upward in immune cells. Because RT-PCR gives relative values, MMP9 protein levels were assayed by immunocytochemistry to have a better understanding of the net change in the concentration of this protease (Figure 1(b)). MDA-MB-231 cells exhibit borderline detectable levels of MMP9 either after single culture or when cocultured with promonocytes. On the other hand, promonocytes had a basal (3D) level of protein (2390 IOD/100 cells) that was increased 3-fold when cocultured with MDA-MB-231 cells (7957 IOD/100 cells).  Figure 1(c) shows images of the levels of MMP9 in promonocytes at the different culturing conditions.

### 3.2. Epithelial and Immune Cell Communication Triggers ECM Degradation

**Figure 2:**
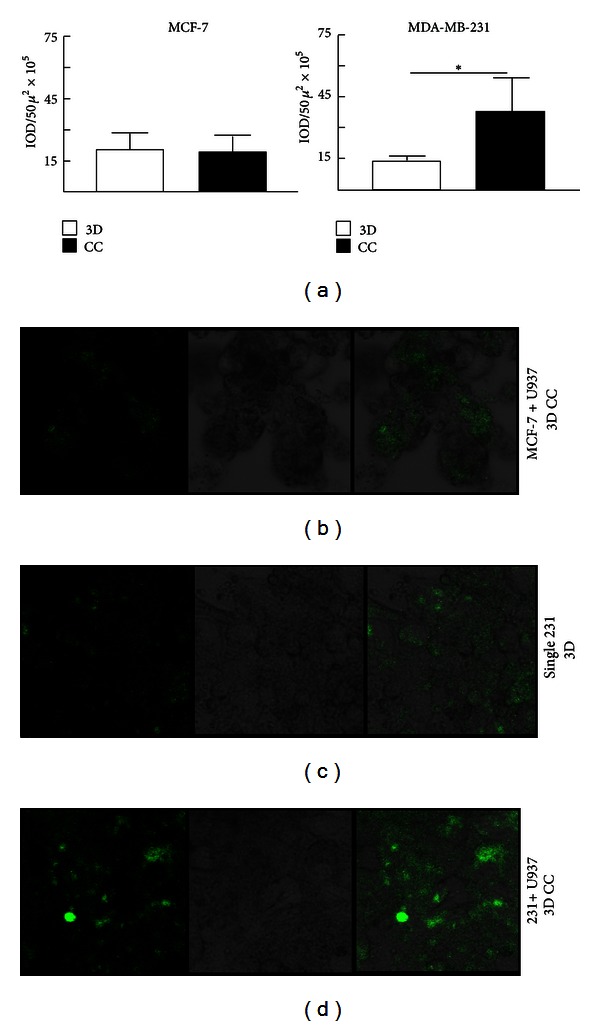
Analysis of extracellular matrix degradation. Cells were cultured in a mix of Matrigel and fluorescently labeled collagen IV, and intensity of fluorescence was measured as an indication of ECM degradation promoted by secreted proteases. (a) Quantification of the release of fluorescence in individual cultures (3D) and in cocultures (CC) between either MCF-7 or MDA-MB-231 and U937 cells. The Bar with asterisk indicates a statistical significant difference (*P* < 0.05). (b–d) Representative images of fluorescence of collagen degradation (left panels), optical images of cells in culture (middle panels), and the merging of both images (right panels) of MCF-7 and U937 cocultures (b), MDA-MB-231 single 3D cultures (c), and MDA-MB-231 and U937 cocultures (d). (b–d): Optical magnification 200x.

Of the three genes found with significantly augmented expression in cocultures, two were proteases (*MMP1* and *MMP9*), whose elevated expressions in the tumor microenvironment are associated with tumor cell invasion through increased degradation of ECM components. To address whether the changes in protease expression affected ECM degradation and whether this effect is depending on cell to cell interactions, cells were cocultured in a matrix of Matrigel containing fluorescent labeled collagen IV. In this system, green-fluorescent peptides released as a result of collagen IV degradation are directly proportional to the proteases secreted by the cell lines in culture [35, 41]. Cells individually or in BRC/promonocyte pairs were cultured for 5 days and the fluorescence emission was quantified; data is presented in Figure 2(a) and a typical example of the fluorescence emitted in the cultures in Figures 2(b)–2(d). Fluorescent levels of single cultures of MCF-7 and MDA-MB-231 cells were 20.2 × 10^5^ and 13.3 × 10^5^ IOD/50*μ* ^2^, respectively. When cells MCF-7 and U-937 were cocultured, there were not significant changes in fluorescence emission (19.9 × 10^5^ IOD/50*μ* ^2^). However, when the highly aggressive BRC cells were cocultured with the promonocytes, there was an additional 2.84-fold increase (coculture 37.8 × 10^5^ IOD/50*μ* ^2^) supporting our initial observation about the increased protease expression triggered by interactions between aggressive tumor epithelial cells and promonocytes.

**Figure 3:**
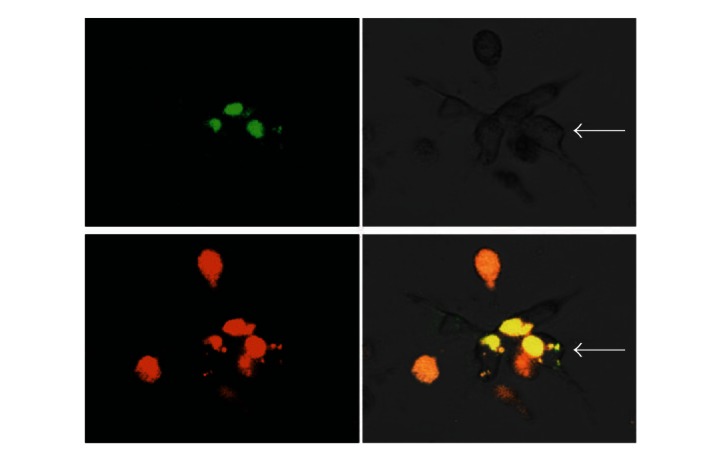
Colocalization of MDA-MB-231 and U937 cells with areas of ECM degradation. Confocal microscopy representative microphotographs of MDA-MB-231 and U937 cocultures. Collagen degradation (upper left panel), optical image of an MDA-MB-231 cell conglomerate (upper right panel), U937 cells labeled with CellTracker Orange (lower left panel), and the merge of all images (lower right panel). The epithelial morphology of MDA-MB-231 cells is pointed with a white arrow. Optical magnification 200x.

In the 3D coculture system, both cellular types were placed in different layers of Matrigel [31, 35]. To have a better understanding about the types of interactions between both cellular lineages promoted in coculture conditions, areas of greater collagen degradation were searched by confocal microscopy. We considered that if protease secretion and therefore fluorescence emission resulted from direct cell to cell interactions, highly green-fluorescent regions should correlate with areas in which both epithelial and immune cells were in close contact; otherwise, a model of communication through soluble factors would be favored. For this analysis, promonocytes were stained with CellTracker Orange to be recognized in culture; the highly aggressive BRC cells were recognized by their epithelial morphology, and areas enriched by both collagen degradation and promonocytes would be yellow. Images were taken of serial slices at a 2-micras distance for a 3D reconstruction of cocultures at different time points (Figure 3 shows an example of fluorescent emission at day 5 of culture). We found that at time 0, the cells were round, individually isolated and no collagen degradation was detected. After 48 h, the highly aggressive BRC cells acquired their characteristic elongated morphology and were grouped in irregularly formed conglomerates (also seen in Figure 2(c)), and cultures already presented proteolytic activity. At day 5, proteolysis was increased and some BRC cells were in close contact with the promonocytes. Figure 3 shows an example in which a conglomerate of BRC cells colocalizes with a few promonocytes. This image also shows collagen degradation in the area of cell colocalization; however, these points of cell interaction were quantified and it was found that they had a relative low frequency (<1%) related to the areas of collagen degradation. Therefore, this analysis argues for a cell to cell communication mainly mediated by soluble factors.

### 3.3. Promonocytes Upregulate COX2 and PGE2 Proteins In Response To Coculturing with Epithelial Tumor Cells

**Figure 4:**
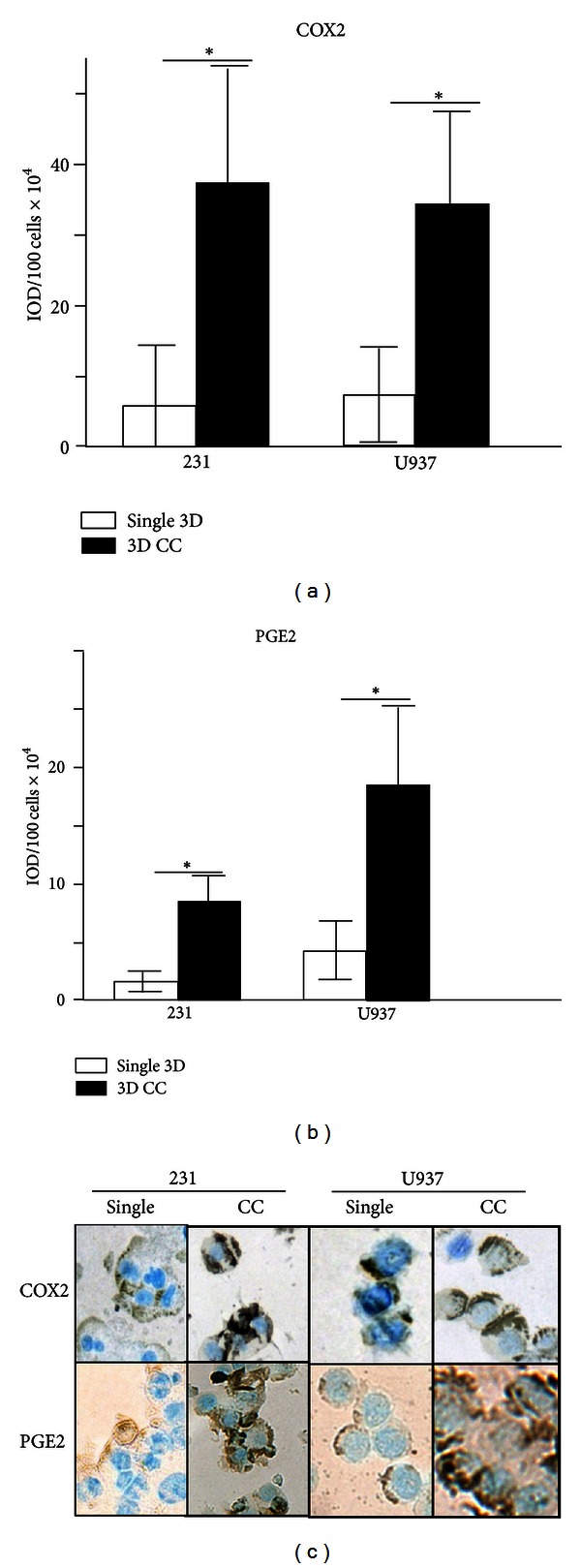
Protein levels of cyclooxygenase 2 and prostaglandin E2. Densitometric analysis of the immunocytochemical detection of COX2 (a) and PGE2 (b) in single MDA-MB-231 and U937 or combined cultures of both cells (CC). Bars with asterisks represent comparisons with statistical significance (*P* < 0.05). (c) Representative images of COX2 and PGE2 levels in MDA-MB-231 and U937 cells after single or combined culture (200x optical magnification).

In addition to proteases, one of the greatest changes in gene expression as a result of coculture was observed in the inflammatory gene *COX2*. This is an interesting result since >50% of DCIS and invasive BRCs overexpress the inducible form of *COX2* and this correlates with aggressive BRC [12]. COX2 is an enzyme that catalyzes the synthesis of prostaglandin 2 (PGE2) from arachidonic acid. To confirm our previous observation, cellular levels of COX2 and PGE2 were analyzed by immunocytochemistry in MDA-MB-231 and U937 cells after 3D culturing individually or both cell lines combined.  Figure 4(a) shows that the levels of COX2 in tumor epithelial cells increased when cocultured from 59401 to 372511 IOD/100 cells, while in the promonocytic cell line there is an increase in coculture conditions from 73591 to 342996 IOD/100 cells. The expression of COX2 correlated with that of PGE2; MDA-MB-231 cells in coculture increased the levels of PGE2 from 18223 to 85419 IOD/100 cells, while U937 cells increased from 42846 to 185141 IOD/100 cells in coculture (Figure 4(b)). A representative image of COX2 and PGE2 levels is shown in Figure 4(c). Overall this experiment confirms the increased expression of COX2 mRNA when cell interactions between tumor epithelial and promonocytes are allowed and illustrates how these interactions favor the establishment of a microenvironment in which protumoral inflammatory factors are enriched.

### 3.4. Soluble Factors Promote the Formation of MCF-10A Acini with Transformation-Like Phenotypes

MCF-10A cells have been widely used as a model to elucidate the transforming mechanisms of viral and cellular oncogenes [33, 34, 42]. Since our previous assays support an important contribution for soluble factors to create an inflammatory protumoral microenvironment, we used MCF-10A cells as sensors of protumoral factors present in media of single and cocultured cells. Thus, we addressed how the supernatant could alter the morphology of the MCF-10A acini-like structures, to better understand the cancer process beyond the genetic changes in the tumor cells themselves.

**Figure 5:**
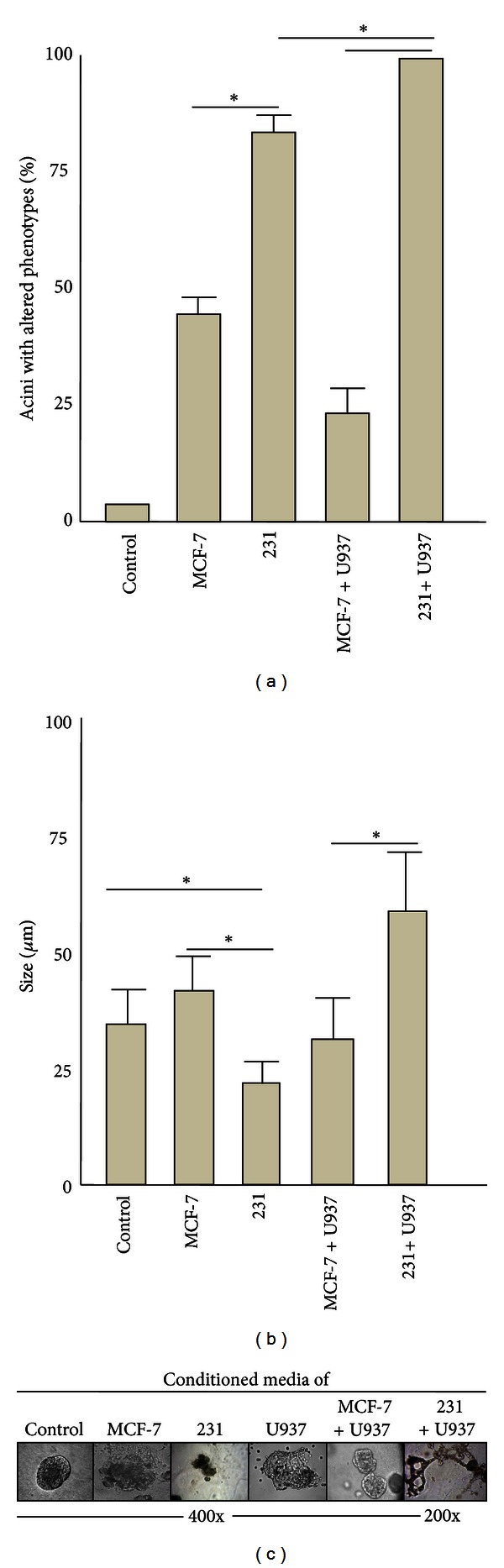
Analysis of MCF-10A acini morphology. MCF-10A acini were formed in a Matrigel-based 3D system in the presence of supernatant from MCF-7 or MDA-MB-231 BRC cells or from cocultures of them and U937 cells. Acini were evaluated based on their shape, structural integrity, size, and presence of a lumen. MCF-10A cells grown in the corresponding dilution of supernatant from an MCF-10A culture were used as control and as a reference of normal acini. (a) Graph representing the average of the frequencies of acini with altered morphology in each culture condition. (b) Graph representing the average size of the acini. (c) Microphotographs of examples of acini that were typically formed under the different culture conditions. Results are from three independent experiments using independent isolates of supernatant. Statistical significance was estimated (*P* < 0.05).

MCF-10A cells were 3D cultured in the presence of supernatants obtained from the poorly and highly aggressive BRC lines or from cocultures of the BRC and promonocyte lines. Glandular acini were monitored daily for 15 days by optical microscopy to assess size (as a measure of cell proliferation), lumen formation (as a measure of resistance to apoptosis), and loss of spherical shape (as a measure of changes in cytoskeletal rearrangements), and results are shown in Figure 5. We observed that most acini grown in supernatant of all cancer cell lines presented aberrant phenotypes, mostly given by increased size and loss of spherical shape. We observed that MCF-10A cells grown in concentrated supernatants formed smaller spheres; it is uncertain whether this is because cells died or there was a proliferative arrest. Acini with aberrant phenotypes were observed at supernatant dilutions of 1 : 4 to 1 : 16. Dilutions 1 : 4 to 1 : 6 of MCF-7 and U937 supernatant gave larger acini, but that of MDA-MB-231 still resulted in smaller acini (Figures 2(b) and 2(c)). MCF-10A cells cultured in the presence of supernatant from a coculture of the highly aggressive BRC cell line and promonocytes exhibited the most aberrant phenotypes, with the largest sizes and the most disorganized structures and without defined lumens. There was a significant difference in the frequency of those aberrant acini between cells grown in supernatant from the highly and poorly aggressive BRC cell lines and also in cocultures of those cells with promonocytes (Figure 5(a)). Some of the acini generated in supernatant of MDA-MB-231 and U937 cocultures exhibited total loss of the normal architecture with elongated shapes often seen as forming networks (Figure 5(c)). In contrast, cocultures of the less aggressive BRC cells generated acini with less deformation, closer to normal colony size and lumen. Overall, this analysis supports that the malignant potential of tumors is importantly mediated by their capacity to engage other cell types to provide protumoral functions to the surrounding environment and this is rendered in great measure by the soluble factors that they secrete.

## 4. Discussion

Tumor aggressiveness results not only from genetic changes in the tumor cell but also from the communication that it establishes with its environment, the stroma of the tumor. All stromal cells participate in the progression of the tumor, probably providing direct cell to cell and cell to ECM interactions, and from the soluble factors they secrete. Tumors are actively recruiting immune cells; however, tumor-associated immune cells often lack immunosurveillance activities, and instead they fulfill protumoral functions (for a comprehensive review see [43]). How tumors favor this immunological switch is not completely understood. In this study, BRC cells with different grades of aggressiveness were cocultured with promonocytes in a Matrigel-based 3D system and upregulation of different genes, often referred to as markers of malignancy, was evaluated.

After the BRC cell lines were allowed to interact with the promonocytes, we observed a tendency to downregulate the genes tested in the BRC lines. Augmented gene expression was mainly found in promonocytes supporting an important flow of information from epithelia to immune cells. It is known that tumor cells secrete CSF-1, a chemotactic factor that actively recruits monocytes to the tumor site [9]. Macrophages normally carry out two functions in tissues: immune effector (phagocytosis, antigen presentation, and release of immune-stimulatory cytokines) and tissue maintenance and regeneration in case of damage. It has been observed that macrophages isolated from tumors had their effector activity suppressed, while presenting increased tissue remodeling activity that favors the growth and metastasis of the tumor [44].

M2 macrophages favor the processes of invasion, intravasation, extravasation, angiogenesis, lymphangiogenesis, and metastasis through secretion of inflammatory mediators, chemokines, growth factors, angiogenic and lymphangiogenic factors, and proteases [45–50]. A meta-analysis found that >80% of studies had a positive correlation between macrophage density and a patient's poor prognosis [4].

It has been proposed that the tumor microenvironment instructs local and arriving macrophages to polarize into M2 types. In physiological conditions, polarization into an M1 phenotype is promoted in a Th1 microenvironment (IFN, GM-CSF, IL12, ROI, RNI, and CXCL10) and into M2 by Th2 cytokines (IL4, IL10, IL13, M-CSF, and CCL2) [51, 52]. An in-deep understanding of the mechanism(s) of tumor-induced polarization into M2 cells and the specific tumor-promoter functions of these cells will provide tools for cancer control.

The results obtained in this study support that important changes are promoted in promonocytes and this type of studies may serve to elucidate the mechanism of immunological switch. Of note, more of the promoted changes are in response to coculture with the most aggressive BRC line. Only *CXCR4* is augmented in response to MCF-7. CXCR4 is the receptor of SDF1/CXCL12, and their interaction promotes increased tumor cell proliferation and survival [53]. CXCR4 also promotes metastasis of BRC cells to distant niches enriched in SDF1 and as such it is an important prognostic marker in BRC [54]. Also, antagonists of CXCR4-SDF1 interactions may have antitumor activity in preclinical studies [55].

No changes in CXCR4 expression were detected in coculture with the most aggressive BRC line; instead *MMP1* and *MMP9* protease genes were significantly upregulated. Proteases are important triggers of cell proliferation, differentiation, matrix remodeling, vascularization, and cell migration. These events take place during organogenesis in normal human development but also during malignant progression. Other proteases functions include the activation of cytokines and growth factors through the excision of their propeptides and release of them through ECM degradation. ECM degradation also facilitates local invasion and metastasis of malignant cells with a migratory phenotype [56].

Our data showed that MCF-7, MDA-MB-231, and U937 cells possess capacity for collagen degradation, but this capacity is augmented when MDA-MB-231 and U937 cells were cocultured. Thus, the BRC aggressive phenotype also coincides with the cell capacity to crosstalk with other lineages. Macrophages have already been described as potent protease producers [57] and MMP9 has been found overexpressed in breast cancer [58, 59] and greatly expressed by M2 macrophages [60]. MMP1 has also been proposed as a marker of progression into BRC [61]. Despite that, our assays do not allow us to conclude that the effect observed is specifically due to these proteases, since other proteases have been implicated in tumor progression and the interplay between them is very complex.

There is direct communication between macrophages and tumor cells importantly mediated by interactions between EGFR-CSF-1 and CXCR4-EGF [62]. One interesting observation in the collagen degradation assays was the presence of U937 cells in contact with the aggregates of MDA-MB-231 cells. It was not possible to determine which of the cell lineages migrated towards the other; however, this migration was not observed in the MCF-7 and U937 cocultures. This cell movement may result from the greater ECM degradation promoted by the MDA-MB-231 and U937 interactions or because of the presence/absence of other important factors in cell chemotaxis.

Another gene significantly augmented in coculture conditions was the inflammatory gene *COX2*. Chronic infection by *Helicobacter pylori* and hepatitis B and C viruses are consistently associated with local chronic inflammation and development of gastric and liver cancers, supporting that chronic inflammatory processes are important triggers of oncogenic lesions [63]. Moreover, inflammatory autoimmune processes, such as Bowel's disease and prostatitis, trigger the appearance of colorectal and prostate cancer, respectively [5]. It has been previously shown that tumor cells induce *COX2* expression in macrophages, which not only favors an inflammatory environment but also increases the synthesis of other protumor factors [64]. *COX2* is upregulated in several cancers in which it has been associated with bad prognosis [65, 66]. In agreement, individuals with excessive blood clotting are frequently treated with periodical amounts of COX2 inhibitors; these individuals have shown lower rates of breast, colon, lung, and prostate cancers [67, 68].

MCF-10A cells have been widely used as a model to elucidate the transforming mechanisms of viral and cellular oncogenes [69–71]. Here, MCF-10A cells were used as sensors of soluble factors with protumoral activity, based on their ability to confer morphological changes to MCF-10A acini, similar to the ones induced by cellular or viral oncogenes. This analysis allowed us to observe the following phenotypes, larger acini with poorly defined lumens and without spherical forms. Normal acini undergo a proliferative arrest around day 10 of culture, resulting in acini of about 35 *μ*M, and larger acini have been related to cells overcoming their proliferative arrest. Also, cells that have lost contact with the ECM-like Matrigel layer die via apoptosis forming the lumen of the acini. Oncogenes turning on apoptosis resistant mechanisms form MCF-10A acini without lumen. Similarly, cells with aberrant cytoskeletal rearrangements lose the spherical shape, and this phenotype often results because of the increased proliferation and loss of the epithelial polarized organization. All of these morphological changes have been considered markers of oncogenic transformation.

Closer to normal acini were observed in MCF-10A cells grown in supernatant from MCF-7 cells, individually or in coculture with U937 cells. On the other hand, the aggressive BRC line in coculture promoted higher frequencies of aberrant acini, which exhibited total loss of the normal architecture, with elongated shapes often seen as forming networks and without defined lumens. Those morphological changes resemble persistent cellular stimulation by oncogene activity, for instance, by constitutive active growth factor receptors. Colonies with a similar morphology are generated by the MDA-MB-231 cells in Matrigel-based cultures (data not shown).

The “seed and soil” hypothesis [72] states that tumor cells released from the primary site are able to generate secondary tumors only in specific organs. This is probably due to the release of soluble factors by the primary tumor that create distant permissive microenvironments for tumor cell colonization, the premetastatic niche [73–75]. Such fertilizing factors affect nontumor tissues in different ways, triggering the recruitment of cooperative populations and tumor cells and promoting angiogenesis in the secondary tumor niche. Those soluble factors may also induce morphological changes similar to the ones promoted in non-tumor cells of the primary site and perhaps also similar to the ones observed here in MCF-10A acini.

## 5. Conclusions

**Figure 6:**
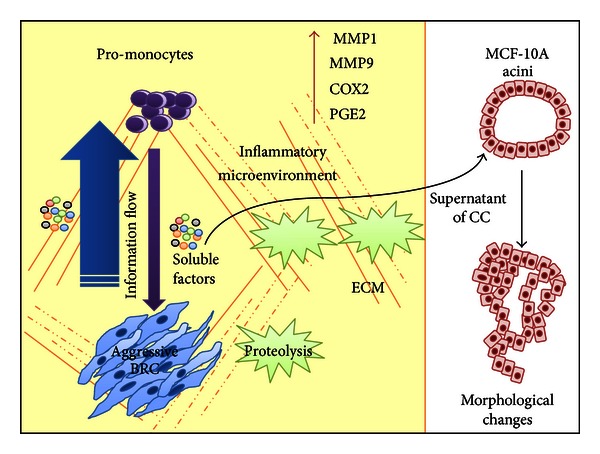
Model of the establishment of an inflammatory protumoral microenvironment. Our data supports that in solid tumors, the expression of genes important for establishing an inflammatory protumoral microenvironment is importantly promoted through cell communication mechanisms between epithelial tumor and other stromal cells, such as promonocytes. The information flows substantially from the BRC cells to the promonocytes, likely in an important extent mediated by soluble factors secreted to the medium. Among the activities promoted is the proteolysis of ECM components. The soluble factors found in supernatant from BRC and promonocyte cocultures were able to trigger morphological changes in MCF-10A acini, similar to changes often seen after oncogene expression (see text for more details). COX2: Cyclooxygenase 2; MMP1,9: metalloproteases 1 and 9; PGE2: prostaglandin E2; ECM: extracellular matrix.

We found a correlation between the level of aggressiveness of the BRC epithelial cell assayed and their capacity to instruct immune cells to modify the expression of genes often reported to favor tumor growth. Among the genes found with augmented expression in promonocytes after interacting with BRC cells were proteases *MMP1* and *MMP9*, which also correlated with functional assays of collagen degradation. Also, the augmented expression of inflammatory factor *COX2* correlated with levels of PGE2. In addition, cocultures of aggressive BRC cells and promonocytes possess a greater ability to trigger in healthy epithelium characteristics of transformed cells, such as an increased proliferation, gross changes at the cytoskeletal level, and absence of lumen (Figure 6). Therefore, these data support that the malignant potential of tumors is not only given by genetic changes in the tumor cell, interactions between tumor and immune cells importantly contribute to the tumor malignant characteristic. They also support the use of 3D coculture systems in which to assay cells or sera isolated from patients to search for markers of tumor aggressiveness. A better understanding of the molecular interactions occurring in the tumor microenvironment would generate better cancer prognostic and treatment tools.
